# Soil fungal communities affect the chemical quality of flue-cured tobacco leaves in Bijie, Southwest China

**DOI:** 10.1038/s41598-022-06593-x

**Published:** 2022-02-18

**Authors:** Mei Wang, Long Zhang, Yi He, Lukuan Huang, Lei Liu, Dan Chen, Anqi Shan, Ying Feng, Xiaoe Yang

**Affiliations:** 1 Key Laboratory of Environment Remediation and Ecological Health, Ministry of Education, College of Environmental and Resource Sciences, Zhejiang University, Hangzhou, 310058 China; 2 Bijie Branch Company of Guizhou Tobacco Company, Guizhou, 551713 China

**Keywords:** Chemical biology, Ecology, Plant sciences, Ecology, Environmental sciences, Solid Earth sciences

## Abstract

Soil microorganisms could affect the quality of tobacco leaves, however, little is known about the association of tobacco chemical components and soil fungal communities. In the present study, the relationship between soil fungi and tobacco quality based on chemical components in Bijie was investigated. The results showed that the total harmony scores (THS) of the analyzed tobacco leaves ranged from 46.55 ± 3.5 to 91.55 ± 2.25. Analyses of chemical components revealed that high contents of nicotine (≥ 1.06%) and sugar (total sugar: ≥ 22.96%, reducing sugar: ≥ 19.62%), as well as low potassium level (≤ 2.68%) were the main factors limiting the quality of flue-cured tobacco leaves. Pearson correlation analysis indicated that soil nitrate, available potassium/phosphorous, and organic matter significantly correlated with tobacco nicotine, potassium, and chloride levels (*p* < 0.05). Besides, the analysis of alpha- and beta-diversity of soil fungal communities implied that fungal structure rather than the richness affected the chemical quality of tobacco. In detail, the relative abundance of *Humicola olivacea* species in soils was positively correlated with the THS of tobaccos (r = 0.52, *p* < 0.05). Moreover, the species including *Mortierella alpina*, *Mortierella hyalina*, *Tausonia pullulan*, and *Humicola olivacea* were negatively correlated with tobacco sugar (r ≤  − 0.45, *p* < 0.05) while, *Codinaea acaciae* and *Saitozyma podzolica* species were negatively correlated with tobacco nicotine (r ≤  − 0.51, *p* < 0.05). The present study provides a preliminary basis for utilizing fungal species in soils to improve the chemical quality of tobacco in the studied area.

## Introduction

China is the largest producer of tobacco (*Nicotiana tabacum* L.) in the world^1^, however, China’s tobacco industry urgently needs to improve the quality of tobacco leaves since the locally cultivated tobacco could not meet the needs of cigarette industry^2^. The quality of flue-cured tobacco includes appearance and smoking characteristics, as well as chemical composition^2,3^. Previously, it is reported that the analysis of smoking and appearance quality of tobacco leaves was greatly dependent on individual judgment, while chemical components were evaluated using scientific apparatus^4^ which showed that the chemical properties of tobacco leaves were consistent with good smoking quality^5^. Therefore, chemical constitutes were the appropriate way to certify the quality of flue-cured tobacco among the three series indexes considering that these contents reflected the taste and aroma of tobacco leaves^6–8^. The tobacco quality is mainly influenced by the climate, soil properties, and microorganism as the climatic factors were reported to be the main factor influencing the tobacco quality in Yunnan Province, Southwest China^9^. Moreover, it is nearly impossible to upgrade the tobacco quality by changing the environmental conditions on a broad scale, but it can be effectively improved by changing the soil properties and microbiota^1,10^. Previous studies have focused on the improved tobacco quality by using biochar products and fertilizers^1,4,10,11^, however, to the authors’ knowledge, few studies reported the relationship between soil microbial communities and tobacco quality through the on-field investigations and experiments.

As the key component of farmland ecosystems, soil microbes play an important role in ecosystem functions and biogeochemical cycles^12–14^, and are considered more sensitive to environmental change than plants^15^. Soil microbes were associated with the changes in natural environments and soil properties, which ultimately affected the quality of crops^16^. A number of studies have reported that soil properties have distinct effects on the biomass and structure of soil microbes, as well as the quality and yield of tobacco^17–19^. For instance, it was stated that soil available phosphorous (AvP) and potassium (AvK) were the main soil factors shifting the structure of fungal communities in soil under continuous cropping of tobacco^19^. Additionally, plants and soil microbes are inextricably linked and mutually influence one another^14^. As for tobacco plant, it has been noted that its chemical composition correlates with the major microbial physiological groups in the rhizosphere soils^5^. However, the understanding of specific microbial species affecting the quality of tobacco leaves in natural environment is still not clear. Therefore, it is of utmost priority to certify the key species related to tobacco quality, which serves as the foundation of exploring microbial resources for tobacco quality improvement.

Fungi play a vital role in agroecosystem, as they contribute to soil nutrients circulation, organic material decomposition, and the health and growth of crop^20,21^. Soil fungi can be divided into harmful or beneficial groups according to their functions in agroecosystem; the harmful fungus can infect crops and lead plant diseases, while the beneficial groups can suppress pathogenic fungi and benefits the crop growth^22,23^. According to the statistics, more than ten kinds of diseases caused by bacterial and viral pathogen were detected in tobacco cultivation, while more than forty diseases triggered by fungus were identified in China^24^. Although, previous researches have focused on the abundance and diversity of soil fungi in continuous cropping of flue-cured tobacco in natural environment^19^, research underlying the relationship between soil fungal communities and tobacco quality, especially in the natural environment, have not yet been fully elucidated. Therefore, this study focused on the impact of soil fungal communities on tobacco quality in a main district of southwest China by planting flue-cured tobacco.

Bijie City is not only a suitable area for producing high-quality of flue-cured tobacco, but also one of the top ten high-quality tobacco technology demonstration areas in China^25^. Thus, Bijie was selected as the research district for this study on relationship of soil fungi and chemical quality of flue-cured tobacco leaves. The present study aimed to (i) understand the chemical quality of the flue-cured tobacco leaves; (ii) investigate the relationship between soil fungal communities and tobacco chemical quality; and (iii) select the key fungal species that contribute to the tobacco quality according to ITS1 gene analysis in Bijie area. The findings would serve as a foundation for utilizing fungal species in soils to improve the chemical quality of tobacco in the research area.

## Results

The analyzed indexes of chemical quality of tobacco leaves include the concentrations of nicotine, total nitrogen, reducing sugar, total sugar, potassium, chlorine, and starch, as well as the ratio of reducing sugar to nicotine (RSN), nitrogen to nicotine (RNN), and potassium to chloride (RKCl). As shown in Table 1, the nicotine content of all samples ranged from 1.06 ± 0.089% to 4.38 ± 0.220%, and good-quality tobacco leaves accounted for 75% of the total samples with a nicotine content of 1.5% to 3.5%. Except for the leaves collected from four sites (labeled as S2, S3, S8, and S20, Table S1), the total nitrogen contents of all samples lied between 1.5% and 3.5%. The reducing sugar of the leaves fluctuated between 19.62 ± 0.99% and 31.77 ± 1.17%, and the total sugar contents varied from 22.96 ± 0.93% to 35.73 ± 1.56%. The potassium level in the tobacco leaves ranged from 1.07 ± 0.005% to 2.68 ± 0.016% (Table 1), while only three samples (S8, S12, and S14) met the standard of high-quality tobacco with the contents over 2%. The chloride level of almost all samples (except S15), less than 1%, were acceptable for cigarette production. Moreover, the starch level of all the studied samples depicted the variation of 2.10 ± 0.174% to 8.13 ± 0.181% (Table 1) with only a quarter of the samples were suitable for cigarette production with the content ranges from 4% to 5%.

**Table 1:** Chemical constituents of the C_3_F (9th to 14th leaf position) tobacco leaves.

Sample ID	Nicotine%	Total nitrogen%	Reducing sugar%	Total sugar%	Chloride%	Starch%	Potassium%
S1	2.83 ± 0.130	1.62 ± 0.116	28.37 ± 1.28	34.56 ± 1.10	0.095 ± 0.009	4.60 ± 0.183	1.62 ± 0.011
S2	1.86 ± 0.092	1.48 ± 0.088	29.90 ± 1.08	33.21 ± 1.65	0.137 ± 0.013	5.24 ± 0.170	1.72 ± 0.009
S3	2.30 ± 0.152	1.26 ± 0.098	31.58 ± 1.23	35.73 ± 1.56	0.077 ± 0.007	6.56 ± 0.176	1.46 ± 0.013
S4	1.89 ± 0.113	1.54 ± 0.127	26.74 ± 1.52	33.57 ± 1.57	0.089 ± 0.017	6.92 ± 0.183	1.56 ± 0.013
S5	3.35 ± 0.267	1.95 ± 0.097	22.83 ± 1.00	26.00 ± 1.42	0.302 ± 0.008	3.70 ± 0.176	1.96 ± 0.017
S6	3.64 ± 0.211	2.01 ± 0.092	24.89 ± 1.19	28.09 ± 1.57	0.281 ± 0.028	2.99 ± 0.178	1.93 ± 0.012
S7	4.38 ± 0.220	2.19 ± 0.102	23.01 ± 0.62	25.11 ± 1.37	0.104 ± 0.010	3.72 ± 0.180	1.45 ± 0.022
S8	1.06 ± 0.089	1.35 ± 0.135	30.03 ± 1.29	34.03 ± 1.13	0.159 ± 0.007	5.69 ± 0.162	2.12 ± 0.009
S9	1.59 ± 0.080	1.53 ± 0.217	29.08 ± 1.40	35.21 ± 1.23	0.092 ± 0.007	7.40 ± 0.189	1.30 ± 0.018
S10	3.05 ± 0.130	2.17 ± 0.117	21.45 ± 1.19	22.96 ± 0.93	0.203 ± 0.010	4.61 ± 0.177	1.74 ± 0.008
S11	2.51 ± 0.116	1.60 ± 0.125	27.72 ± 0.90	31.81 ± 1.19	0.105 ± 0.009	6.58 ± 0.184	1.30 ± 0.013
S12	3.47 ± 0.136	2.09 ± 0.125	24.48 ± 0.74	31.23 ± 1.19	0.092 ± 0.008	4.73 ± 0.188	2.18 ± 0.015
S13	2.66 ± 0.085	1.54 ± 0.117	23.61 ± 1.01	32.69 ± 1.14	0.168 ± 0.017	8.13 ± 0.181	1.63 ± 0.014
S14	3.56 ± 0.175	2.48 ± 0.217	25.91 ± 1.73	27.02 ± 0.82	0.142 ± 0.008	3.56 ± 0.191	2.68 ± 0.016
S15	2.59 ± 0.123	2.10 ± 0.088	29.73 ± 0.67	31.42 ± 0.78	1.161 ± 0.016	4.04 ± 0.182	1.48 ± 0.014
S16	3.27 ± 0.237	1.80 ± 0.107	31.77 ± 1.17	32.95 ± 1.47	0.239 ± 0.003	5.87 ± 0.185	1.68 ± 0.010
S17	3.83 ± 0.196	2.31 ± 0.143	23.00 ± 1.03	25.63 ± 1.11	0.167 ± 0.009	2.95 ± 0.179	1.07 ± 0.005
S18	2.76 ± 0.173	1.99 ± 0.065	19.62 ± 0.99	25.70 ± 0.66	0.187 ± 0.011	2.10 ± 0.174	1.22 ± 0.003
S19	2.18 ± 0.124	1.67 ± 0.132	27.99 ± 1.23	30.71 ± 0.95	0.477 ± 0.006	4.28 ± 0.167	1.09 ± 0.010
S20	1.79 ± 0.087	1.41 ± 0.095	27.99 ± 1.46	35.44 ± 0.86	0.300 ± 0.008	6.00 ± 0.172	1.22 ± 0.012

**Figure 1:**
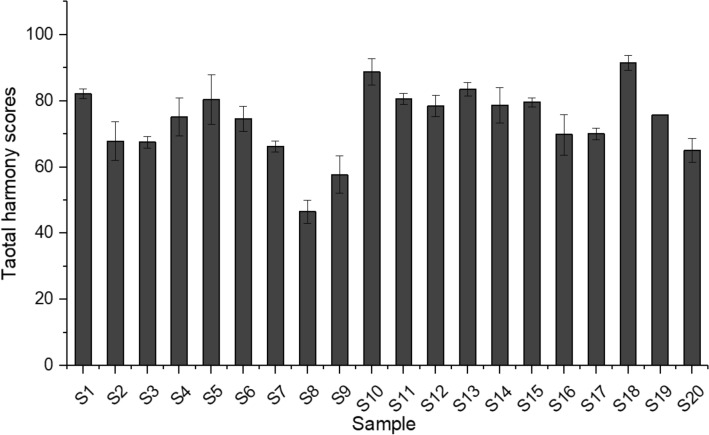
The total harmony scores of all the flue-cured tobacco leaves samples.

Total harmony scores (THS), which reflected the overall chemical quality of flue-cured tobacco leaves, differed significantly across all samples (Fig. 1). The TSH values ranged from 46.55 ± 3.50 to 91.55 ± 2.25 with an average value of 73.99 ± 10.86 across all samples (Fig. 1). High scores of certain indexes of chemical components did not reflect the overall quality of flue-cured tobacco leaves (Table S2). For example, the score of potassium of leaves samples collected from S8 was up to 92.40 ± 0.19, but the THS of the sample was as low as 46.55 ± 3.50 (Table S2).

**Figure 2:**
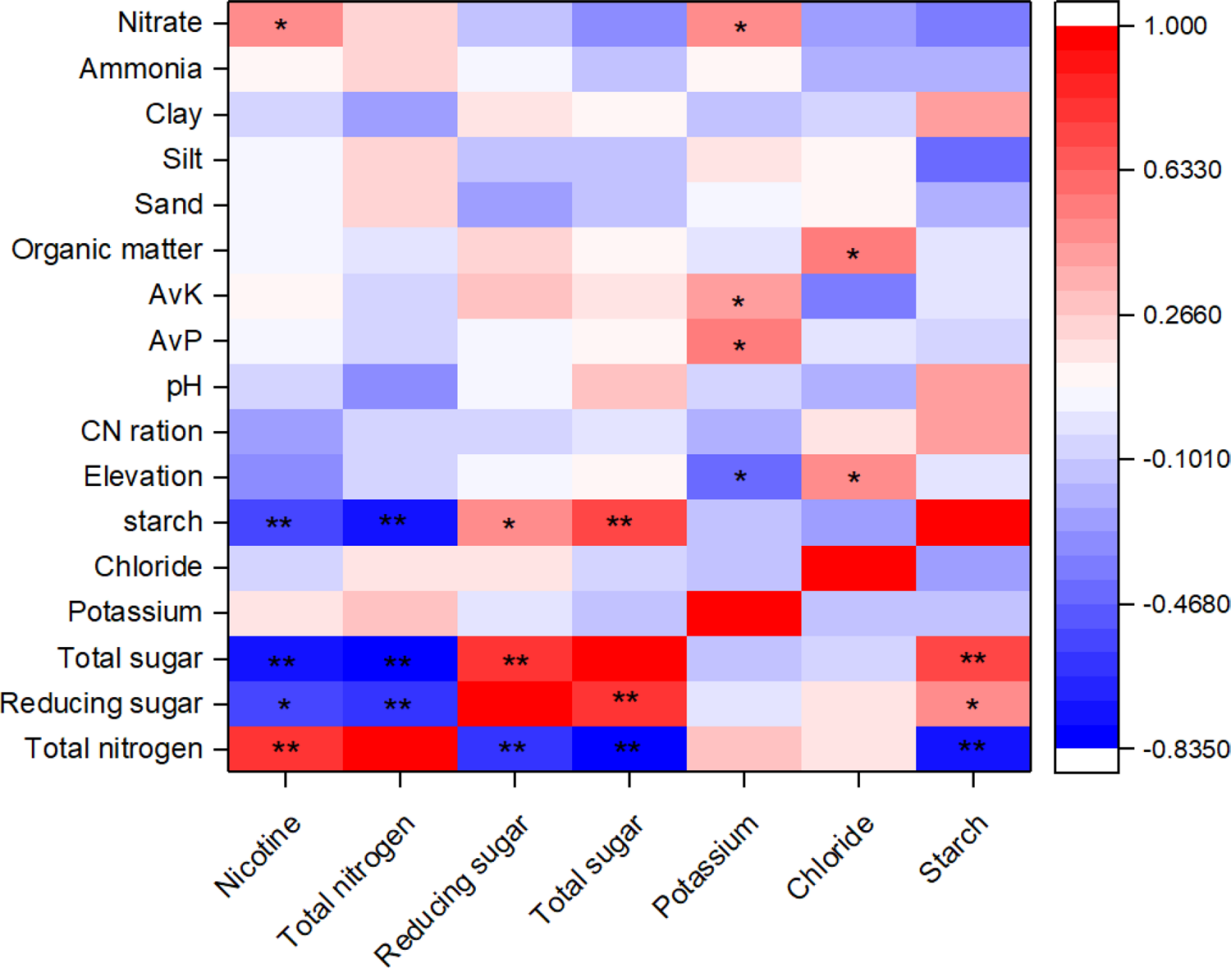
Heat map basing on Pearson correlation coefficients among chemical constituents of the tobacco leaves (n = 20). *AvK* available potassium, *AvP* available phosphorous, *CN ratio* ratio of total carbon and nitrogen concentrations in soil.

Pearson correlation analysis among different parameters of tobacco chemical quality showed a positive correlation between nicotine and total nitrogen (r = 0.815, *p* < 0.01, Fig. 2). Additionally, tobacco sugar levels (include reducing sugar and total sugar) were negatively correlated with both of nicotine and nitrogen concentrations (r <  − 0.551, *p* < 0.01), whereas a strong positive correlation between the reducing sugar and total sugar was observed (r = 0.815, *p* < 0.01, Fig. 2). The starch level was negatively correlated with concentrations of nicotine and total nitrogen (r <  − 0.590, *p* < 0.01), but positively associated with sugar levels (r > 0.509, *p* < 0.01, Fig. 2). However, it was observed that potassium or chloride did not show any significant correlation with the other analyzed components (*p* > 0.05, Fig. 2). The correlations between environmental factors (include elevation and selected soil properties) and tobacco chemical components are visualized in Fig. 2. The results depicted that the elevation of sampling sites showed a positive association with chloride levels of tobacco leaves (r = 0.477, *p* < 0.05), but negatively associated with the potassium content (r =  − 0.463, *p* < 0.05, Fig. 2). The soil AvP (r = 0.516), AvK (r = 0.448), and nitrate (r = 0.500) were positively correlated with potassium level of tobacco leaves (*p* < 0.05, Fig. 2). Moreover, a strong and positive correlation between soil nitrate and plant nicotine level was observed (r = 0.451, *p* < 0.05), but tobacco nicotine had no significant correlation with soil ammonia (*p* > 0.05, Fig. 2). A significant relationship of plant chloride level and soil organic matter content depicted the r = 0.569 and *p* < 0.05 values and are presented in Fig. 2. It was worthy to note that total nitrogen, sugars, and starch levels of tobacco had no significant association with soil properties (*p* > 0.05, Fig. 2).

After pair-end Reads alignment and filtering, 4,146,724 high-quality clean tags were collected across all samples, which were classified into 822 operational taxonomic units (OTUs) using a 97% sequence similarity criterion. The rarefaction curves and Shannon curves tend to be flat (Fig. S1) indicating that the amount of sequencing data is sufficient to reflect the majority of fungal diversity information and the amounts of OTUs reached saturation for all the samples^26^. The range of fungal OTUs (372 ± 37.9 to 501 ± 24.0) and Chao1 values (413 ± 32.2 to 560 ± 61.8, Table 2) indicated that the fungal richness varied greatly across all the soil samples. The Shannon index varied from 4.36 ± 1.97 (S17) to 6.14 ± 0.58 (S15) and did not show any significant difference (except S13–15, 17) among all analyzed samples (*p* > 0.05, Table 2). With a difference in Shannon index, the Faith’s PD values were changed with soil samples within a range of 48.7 ± 1.06 (S16) to 70.7 ± 4.22 (S1, Table 2). All these data indicated the variation in fungal community richness and diversity between the sampling sites. Generally, fungal community with high values of OTUs and Chao obtained high Faith’s PD index, and vice versa (Table 2).

**Table 2:** Summary of OTUs, Chao 1, Shannon, and Faith’s PD indexes based on relative abundance of fungal sequences at different sites.

Sample ID	OTUs	Chao1	Shannon	Faith’s PD
S1	492 ± 35.6ab	560 ± 61.8a	5.03 ± 0.54ab	70.7 ± 4.22a
S2	442 ± 22.1a–f	497 ± 32.0a–e	5.49 ± 0.05ab	63.0 ± 2.36b–d
S3	382 ± 45.9fg	447 ± 44.7d–f	4.66 ± 1.20ab	56.1 ± 4.88e–g
S4	372 ± 37.9g	413 ± 32.2f	5.26 ± 0.92ab	52.7 ± 2.92gh
S5	472 ± 26.5a–c	535 ± 37.5a–c	5.24 ± 0.10ab	69.1 ± 3.64ab
S6	381 ± 61.5fg	434 ± 57.6ef	4.55 ± 1.21ab	56.6 ± 7.07d–g
S7	394 ± 26.9e–g	463 ± 22.9c–f	5.41 ± 0.60ab	58.4 ± 0.65c–g
S8	375 ± 16.3g	425 ± 15.0ef	4.75 ± 0.58ab	56.8 ± 1.24d–g
S9	423 ± 20.2c–g	484 ± 5.58b–f	5.89 ± 0.02ab	63.4 ± 2.17b–d
S10	402 ± 14.6d–g	470 ± 22.0c–f	5.76 ± 0.32ab	58.4 ± 2.95c–g
S11	462 ± 12.8a–d	519 ± 20.1a–d	5.66 ± 0.69ab	59.9 ± 2.77c–f
S12	469 ± 22.3a–c	552 ± 35.8ab	5.13 ± 0.64ab	60.4 ± 1.34c–e
S13	438 ± 16.4b–f	496 ± 26.5a–e	6.08 ± 0.03a	57.0 ± 1.80d–g
S14	447 ± 43.9a–e	520 ± 37.9a–d	4.39 ± 1.48b	62.0 ± 3.50c–e
S15	501 ± 24.0a	553 ± 26.7ab	6.14 ± 0.58a	64.5 ± 1.66bc
S16	375 ± 6.66g	428 ± 33.2ef	4.92 ± 0.35ab	48.7 ± 1.06h
S17	413 ± 54.1c–g	494 ± 62.7a–e	4.36 ± 1.97b	55.6 ± 5.87e–g
S18	433 ± 6.81b–g	476 ± 7.85c–f	5.85 ± 0.16ab	54.3 ± 1.67f–h
S19	413 ± 15.4c–g	509 ± 34.1a–d	4.72 ± 0.27ab	57.7 ± 1.17d–g
S20	442 ± 39.6a–f	509 ± 53.9a–d	5.25 ± 1.20ab	59.1 ± 6.07c–g

Different letters represent statistical significance at *p* < 0.05.

**Figure 3:**
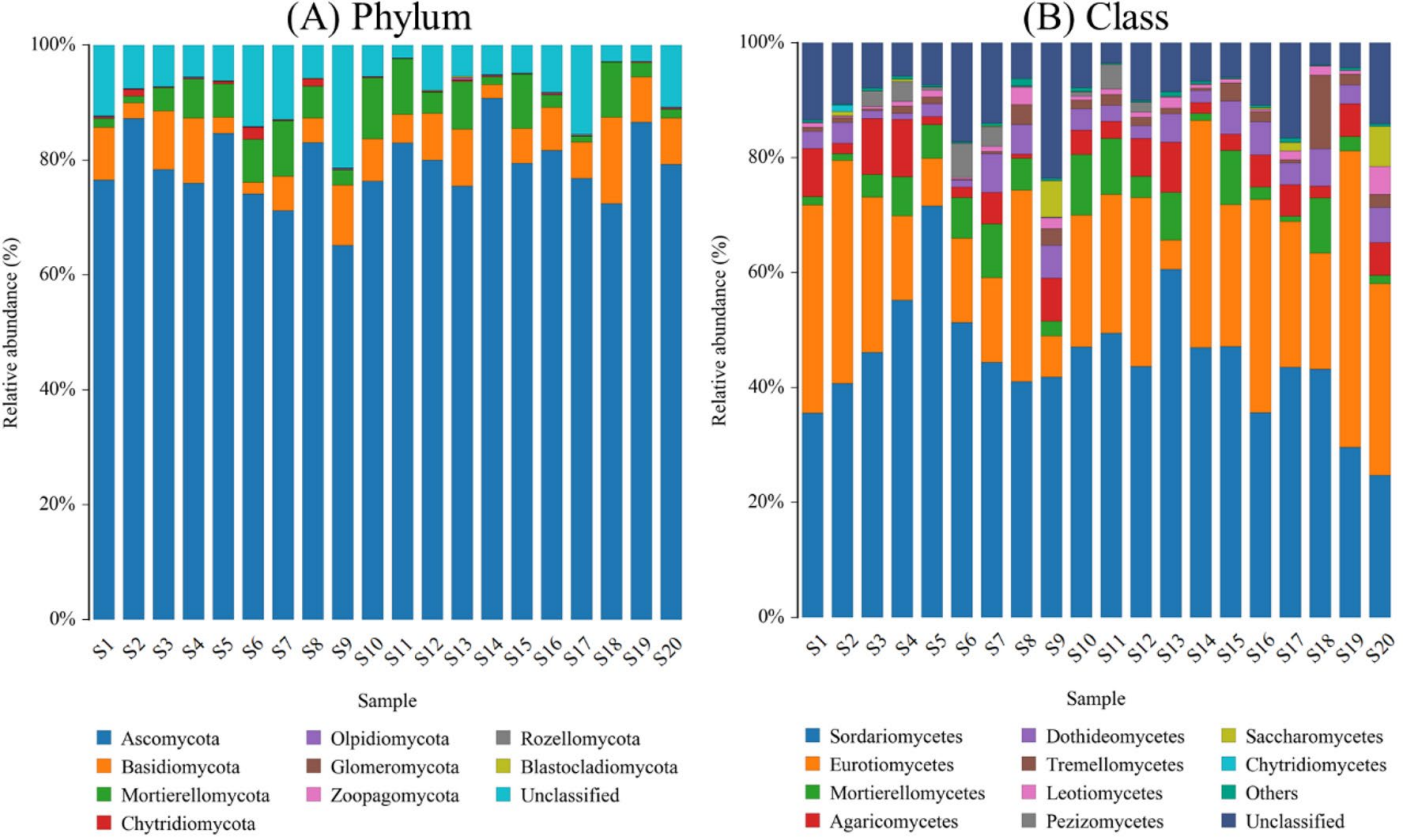
Relative abundance of dominant phyla (**A**) and classes (**B**) of fungi in the soil.

**Figure 4:**
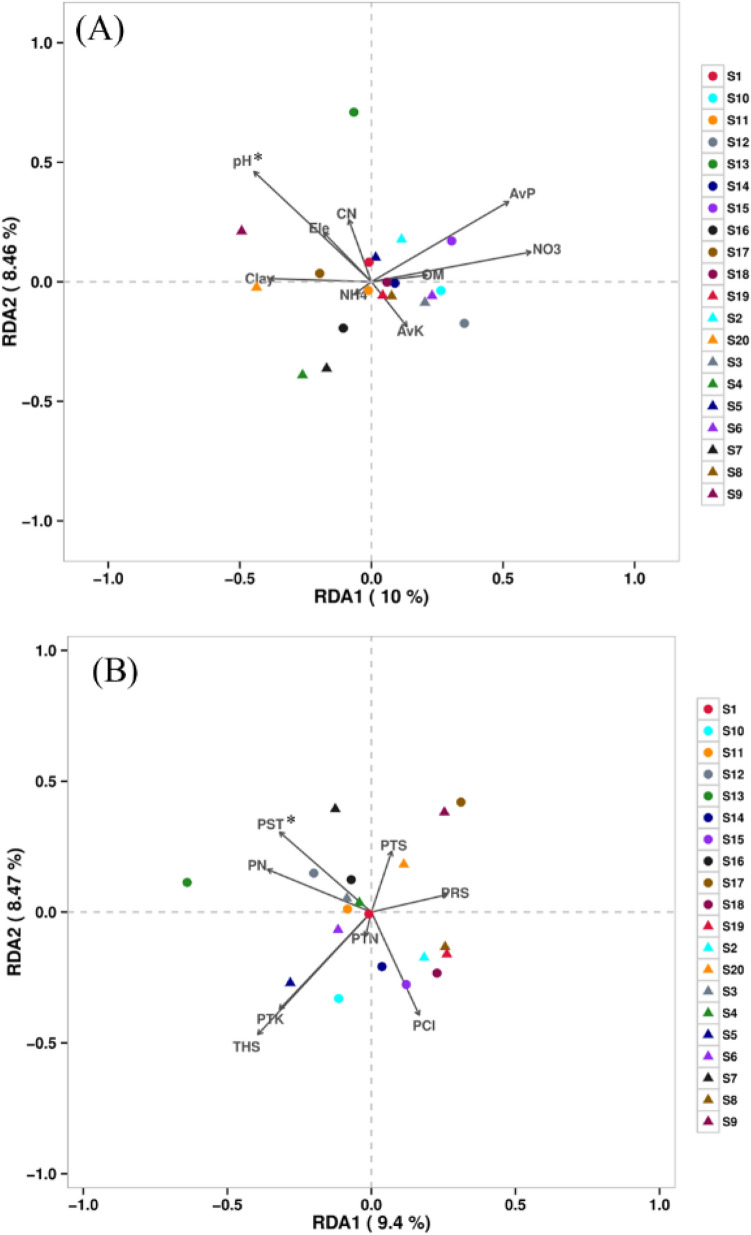
Redundancy analysis (RDA) of soil fungal communities with selected environmental factors (**A**) and chemical components of flue-cured tobacco leaves (**B**). The environmental variables include *Ele* the elevation of the sampling site, *pH* soil pH, *CN* soil C:N ratio, *Clay* soil clay content, *OM* soil organic matter content, *NH4* soil ammonia content, *NO3* soil nitrate content, *AvK* available potassium content, *AvP* soil available phosphorus content. Tobacco quality variables includes *PN* tobacco nicotine, *PTN* tobacco nitrogen, *PRS* tobacco reducing sugar, *PTS* tobacco total sugar, *PST* tobacco starch, *PTK* tobacco potassium, *PCl* tobacco chloride, *THS* total harmony scores of flue-cured tobacco leaves.

**Figure 5:**
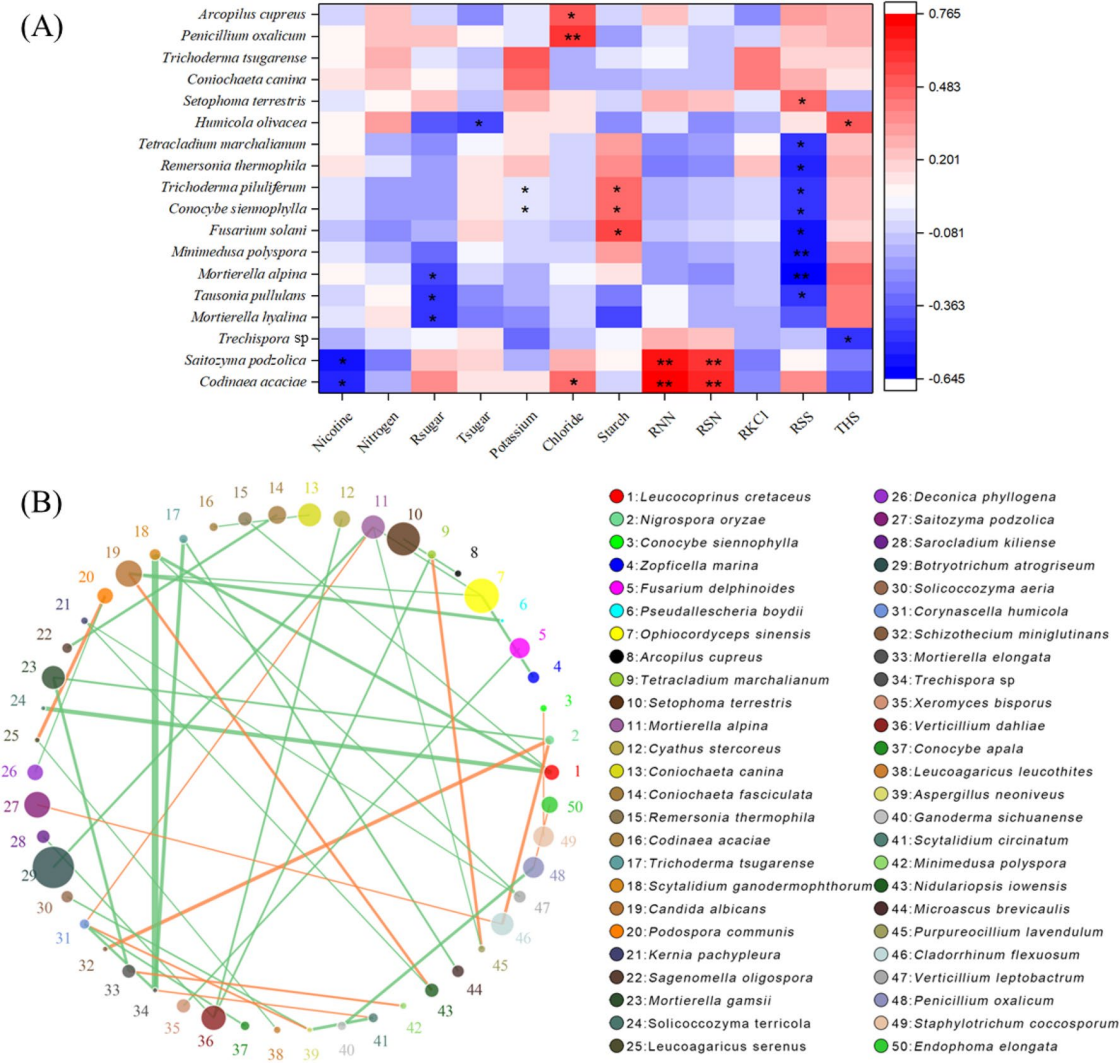
Relationships of fungal richness at species level with quality of tobacco leaves (**A**) and the network at species level (**B**). Tobacco chemical components includes nicotine, nitrogen, reducing sugar (Rsugar), total sugar (Tsugar), potassium, starch, ratio of nitrogen to nicotine (RNN), ratio of reducing sugar to nicotine (RSN), ratio of potassium to chloride (RKCl), ratio of reducing sugar to total sugar (RSS), and total harmony scores (THS). The analyses were constructed using the top 60 most abundant species of soil fungal community, the displayed in the picture was at the significant of *p* < 0.05 level with r ≥ 0.2. Circles represents species, size of circle represents the abundance. The edges represent the correlation between the two species, the thickness of the edge represents the strength of the correlation, and the color of the line: orange represents the positive correlation, while green represents the negative correlation.

The dominant relative abundances of ten phyla and classes of soil fungi were selected to generate the column diagrams of different sampling sites (Fig. 3). Across all the samples, at phylum level, the most abundant phylum was *Ascomycota* with an average relative abundance of 78.91%, followed by *Basidiomycota* (average of 7.12%) and *Mortierellomycota* (average of 5.23%) (Fig. 3A). However, at the class level, the most abundant classes were *Sordariomycetes*, *Eurotiomycetes*, *Mortierellomycetes*, *Agaricomycetes*, and *Dothideomycetes* with average relative abundance of 44.97%, 25.41%, 5.17%, and 4.95% respectively (Fig. 3B). In order to analyze the correlation of fungal species with elevation, soil properties, and tobacco quality, the redundancy analysis (RDA) was performed. As for elevation and soil properties, the results showed that the first and second axis explained 10.00% and 8.47% of the variation of fungal community in soils (Fig. 4A). In terms of tobacco chemical quality, the first two RDA axes totally explained 17.87% of the variation in fungal community (Fig. 4B). It was observed that both the environmental factors and tobacco plants have influenced the fungal communities in soils, with the soil pH (r = 0.358, *p* < 0.05) and starch contents of tobacco leaves (r = 0.348, *p* < 0.05) being the most significant variables associated with soil fungal composition (Fig. 4). Moreover, a comprehensive and detailed correlation analysis between tobacco chemical quality and the top 60 most abundant fungal species was also studied (*p* < 0.05, Fig. 5A). The results showed that only 18 fungal species had significant associations with tobacco chemical quality. Except for total nitrogen, potassium, and RKCl, the other indexes of tobacco chemical quality were significantly related with certain soil fungal species (*p* < 0.05, Fig. 5A). Importantly, the THS of tobacco leaves was positively correlated with *Humicola olivacea* species (r = 0.525, *p* < 0.05), but negatively correlated with *Trechispora* sp species in the soils (r =  − 0.471, *p* < 0.05, Fig. 5A). Meanwhile, *Trechispora* sp slightly related with *Scytalidium circinatum* (r = 0.208), but negatively correlated with *Corynascella humicola* (r =  − 0.234), *Trichoderma tsugarense* (r =  − 0.261), and *Scytalidium ganodermophthorum* (r =  − 0.343, *p* < 0.05, Fig. 5B). However, no relationship between *Humicola olivacea* and the other species was observed (Fig. 5B). Besides, plant nicotine negatively related with soil *Saitozyma podzolica* (r =  − 0.555, *p* < 0.05) and *Codinaea acaciae* (r =  − 0.513, *p* < 0.05), while RNN and RSN were positively correlated with these two species (r ≥ 0.604, *p* < 0.05, Fig. 5A). It is worth noting that tobacco sugars had a negative correlation with *Mortierella alpina*, *Mortierella hyalina*, *Tausonia pullulans*, and *Humicola olivacea* species in soils (r ≤  − 0.445, *p* < 0.05, Fig. 5A).

## Discussion

In the present study, the chloride levels in were found to be acceptable in a number of samples but were lower than the range of high-quality tobacco leaves (0.4–0.8%)^27^. It is possible to coordinate the chlorine content in flue-cured tobacco leaves through manure organic fertilizer or biochar^28^, and similarly in our study, a positive correlation between chlorine level in the leaves and soil OM content was detected (Fig. 3). Besides, chloride level in tobacco samples showed a positive correlation with altitude (r = 0.477, *p* < 0.05, Fig. 3), and this was coincided with the result that climate was a main factor which influence the chemical components of tobacco^9^. The high sugar contents of the flue-cured tobacco leaves described in this work are concordant with previous studies that reported reducing sugars and total sugar levels ranges of 13.62–32.60% and 18.16–40.81%, respectively^27^. The results also indicated that the reducing sugar and total sugar contents were higher than the standard (16–18% and 18–22%, respectively) for good-quality of tobacco^27^. For decades, high sugar content was considered as a vital factor which affects the tobacco quality in Bijie City, and this might be owed to the climatic conditions of this area. For example, in areas with high elevation, a significant temperature difference between day and night favored the sugar buildup in plants^29^. However, no significant correlation between tobacco sugar and elevation (*p* > 0.05) might be due to the complex and changeable soil properties across all the samples (Table S1). In this study, the low potassium level in almost all samples corresponds to a previous research that tobacco potassium content is typically lower than 2% in China^30^, results in restricting the improvement of tobacco quality^6^. The results of our study showed correlations between the tobacco potassium and soil AvK, nitrate, AvP, as well as elevation (Fig. 3), indicates that both of soil nutrition and climatic environment have an effect on the potassium flow within tobacco plants. Therefore, it is acceptable to increase the potassium contents of tobacco by fertilization and improving the microbiota which aid in nutrients absorption^28^.

Nitrogen is not only an essential element for the high yield and good quality of tobacco^31^, but also correlates with the nicotine formation in tobacco leaves^32^. The present investigation established this result by demonstrating a substantial correlation between tobacco nitrogen concentration and nicotine content (r > 0.58, *p* < 0.01, Table 2). The accumulation and reduction of nicotine in tobacco is affected by nitrogen, and importantly, the effects depends on the form of applied nitrogen^32^. Nicotine in tobacco leaves positively relates with soil nitrate rather than ammonia or total nitrogen in soils (Fig. 3), which was partly consistent with a previous report that nitrate was recommended for elevated tobacco production in contrast to that ammonium reduced the yield and leaf quality of tobacco^11^. The nicotine contents (1.06–4.38%) were in line with the investigation of 2012 where the nicotine contents ranged from 0.95% to 4.81%^27^. Lowering the nicotine content in tobacco plants was a primarily measure for the reduction of nicotine in tobacco products^33^. However, despite that the 75% of the total samples meet the requirement of high-quality tobacco based on the nicotine level; it is of dire need to reduce its content in flue-cured tobacco leaves in the study area. The positive correlation between tobacco nicotine and soil nitrate but not with other soil properties (Fig. 2) indicates that it might be impossible to decrease tobacco nicotine levels through fertilization.

The average THS (73.99 ± 10.86) in the present study was lower than the results of a previous report with the average value higher than 81.15^27^, indicating the reduction in the quality of flue-cured tobacco leaves as compared with 2012. These probably because of the increment of fertilization which usually results in an increase in yield, but a decrease in quality^34^. Totally, an improvement in the chemical quality of tobacco could be done by reducing the contents of nicotine and sugar as well as increasing the potassium level in tobacco leaves. As for sugar level, it is difficult to improve the tobacco quality by adjusting the soil properties (Fig. 2). Enhancing potassium level in tobacco leaves by applying potassium and phosphorous fertilizers was preferable than nitrate fertilizer which would increase tobacco nicotine content (Fig. 2). The application of fertilizers over the years has led to an increase in potassium and phosphorus contents in soils^35^, however, the amount of potassium that can be absorbed by tobacco plant is still limited. Moreover, a part of nicotine in tobacco roots is released into soil, which could affect AvP, AvK, and the proliferation of microorganisms in soil^36^. In turn, microbes in soils may influence the growth of tobacco plants, and affects the tobacco quality^37^. It was reported earlier that the microorganisms boosted the soil fertility and plant nutrition^38^, and was an inexpensive and eco-friendly approach to improve the tobacco quality. Considering that microbial community might change with the local environment^39^ and tobacco disease caused by fungal pathogen was more prevalent than that by bacterial pathogen, it is essential to understand the key fungal communities correlated with tobacco quality.

With single disturbance, the changes of soil fungal diversity and communities are determinable^26,40^. In the present study, however, complex environmental conditions induce no relationship between tobacco chemical components and soil fungal richness (Fig. S2). This was partly consistent with the result of a previous report that no close relationship was found between chemical composition of tobacco and soil fungal population^5^. The RDA analysis showed that elevation and selected soil factors totally explained 18.47% of the variation of soil fungal community while tobacco chemical constituents explained 17.87% of the variation (Fig. 4). The explained variation of the axes for fungal community in soils was quite low maybe due to the absence of such determinants in the current study including the temperature, rainfall, soil type, and the other vital compounds in tobacco plant, which influence the soil microbial communities^9^. Even so, the soil pH was a significant variation affected soil fungal communities (*p* < 0.05, Fig. 4) which was also an important factor for the growth and nutrients absorption of plant.

The positive relation between THS of tobacco leaves and soil *Humicola olivacea* species was probably because of the metabolites produced by the fungal species^41^. In the current study, species of *Humicola olivacea* showed no association with other fungal species in soils (Fig. 5B). Therefore, optimizing the chemical compatibility of flue-cured tobacco by improving soil fungal communities would pay consideration in the enhancement of *Humicola olivacea* species. According to the correlation between tobacco nicotine and soil fungal species, as well as the relations among fungal species in soil (Fig. 5), enhancing *Saitozyma podzolica* and *Codinaea acaciae* species in soils might be an appropriate way to decrease tobacco nicotine content in the study area. It was noteworthy that *Saitozyma podzolica* species was beneficial for the degradation of plant cellulose and might be useful for sustainable lipid production in industrial applications^42^. Thus, it should be feasible to reduce the tobacco nicotine levels by improving soil *Saitozyma podzolica* species in Bijie area. The sugars in flue-cured tobacco leaves in Bijie are frequently in high levels^27^. It was reported that measures to decrease the sugar contents in flue-cured tobacco leaves by using biochar, chemical fertilizers, and baking were limited^43,44^. However, it is possible to decrease tobacco sugar contents by improving soil fungal communities, while plant sugar was negatively correlated with *Mortierella alpina*, *Mortierella hyalina*, *Tausonia pullulans*, and *Humicola olivacea* species in soil (r ≤  − 0.445, *p* < 0.05, Fig. 5A). Considering the weak correlations of these four fungal species with the other fungal species in soil (0.111 ≤ r ≤ 0.230, Fig. 5B), their optimal application in soils should not disturb the balance of soil microecology.

It is of utmost attention that increasing the potassium levels in tobacco leaves is a vital but difficult work in tobacco planting in China^6,43^. Despite that the tobacco potassium had no relationship with soil fungal communities at the species level, its positive association with soil *Trichoderma* genus (r = 0.525, *p* < 0.05) would make it possible in improving the potassium content by biotechnology. *Trichoderma* is a natural and ubiquitous fungal genus in soils that produce antifungal metabolites which may compete, inhibit, or cause lysis of several structures of plant fungal pathogens^45^. Besides, this fungal community has ability to influence the composition of plants, and was resistant to some manmade pollutants^46^. Moreover, *Trichoderma*-based products are commercially marketed worldwide as bio-fungicides, plant bio-stimulants, and biological soil amendments^46^. Therefore, it is possible to use *Trichoderma*-based products in soils or tobacco plant to improve soil ecosystem stability and tobacco quality. By combining the soil fungal communities, soil properties and local conditions, it would be possible to improve the chemical quality of tobacco leaves by increasing soil nutrients and adjusting the composition of soil fungal structure.

## Conclusions

In the present study, we explored the relationship between soil fungal communities and chemical quality of flue-cured tobacco leaves in Bijie. Chemical quality of almost all samples was acceptable according to the THS standards, however nicotine, sugar, and potassium levels were the main components limiting the quality of flue-cured tobacco leaves. The drivers affecting the tobacco quality were analyzed which elucidated that the elevation and soil properties, including soil nitrate, AvK, AvP and OM, were the vital indexes. As for soil fungal communities, no relationship of fungal richness and chemical quality of the flue-cured tobacco leaves was observed. However, THS of the leaves positively correlated with *Humicola olivacea* and *Trechispora* sp. species in soils, and enrichment of *Humicola olivacea* species in soils helps in improving the chemical quality of tobacco leaves. Besides, it should be feasible to reduce tobacco nicotine by improving soil *Saitozyma podzolica* species, while tobacco potassium content might be increased by using *Trichoderma*-based products in soils. Moreover, the appropriate use of *Mortierella alpina*, *Mortierella hyalina*, *Tausonia pullulans*, and *Humicola olivacea* species in soils may decrease tobacco sugar level with no disturbance to the balance of soil microecology. To sum up, improving the tobacco quality need to combine the nutrients and fungal structure in soil. It is necessary to investigate the interactions between tobacco quality and soil bacterial communities, as well as the use of biofertilizer to stimulate tobacco quality in the future studies.

## Methods

Bijie (105° 36′–106° 43′ E, 26° 21′–27° 46′ N) has a subtropical monsoon climate, with an annual temperature of 13.4 ℃, and the annual precipitation ranges between 849 and 1399 mm^47^. The Bijie’s altitude is from 457 to 2900 m, and the total cultivated area is 364,690 ha, which only account for 13.58% of the total area^48^. The annual acreage and yield of flue-cured tobacco were found to be approximately 5000 ha and 120,000 tons, respectively^49^.

Soil samples were collected at the maturity of tobacco plants in July 2019. For thesis, twenty sampling sites were selected in the tobacco-planting fields of Bijie City, and both of elevation and coordinates of the sampling sites were recorded using a GPS system (Table S1). Sampling tools which include stainless steel shovels were disinfected with 75% alcohol, while sterile polythene plastic bags were used for soil samples collection. Three samples were collected from each site which contained five upper 10-cm soil cores randomly distributed in the farmland. The collected soil samples were stored in ice container and transported to the laboratory immediately after sampling^50^. Plant residues and obvious stones were removed before the samples were sieved < 2 mm. The sieved soil was then divided into two subsamples, one was stored at − 80 ℃ for soil DNA extraction, and the remainder was air-dried for physiochemical analysis^51^.

Tobacco plants were manually harvested 5 times at around 1 week interval by removing 3 to 5 leaves each time, and the leaves were promptly cured in a flue-curing barn for flue-cured tobacco^52^. Then, the cured leaves samples of C_3_F (9th to 14th leaf position), which represent middle leaves were selected for the subsequent analysis^43^. Only one flue-cured tobacco leaves sample (around 2 kg) was selected in October 2019 because the environmental factor and curing process was same for the same farmland. The main veins of the leaves were removed, and the sample was grinded and sieved through the 40 mesh after dried at 40 ℃ before analyzing the chemical components. The total nitrogen, nicotine, potassium, chloride, starch, reducing sugar, and total sugar with three technical replicates were analyzed with continuous flow method. To evaluate the harmony of the chemical components, which reflect the chemical quality of flue-cured tobacco, the contents of reducing sugar, nicotine, total nitrogen, potassium, starch, and RNN, RSN and RKCl were selected for grading the chemical quality of flue-cured tobacco leaves (Table S3)^27,28^. According to the reports, the total harmony scores (THS) = score of nicotine × 0.17 + score of total nitrogen × 0.09 + score of reducing sugar × 0.14 + score of potassium × 0.08 + score of starch × 0.07 + score of RSN × 0.25 + score of RNN × 0.11 + score of RKCl × 0.09. The higher the THS, the better the chemical quality of flue-cured tobacco leaves.

For the analyses of soil properties, the methods commonly used in previous research were employed^53^. Briefly, soil pH was tested by a pH detector (Sartorius PB-10) in a soil–water (1:2.5) solution. Soil OM was determined by the Walkley–Black wet digestion method. Soil AvP and AvK was extracted by NH_4_F-HCl (or NaHCO_3_) and CH_3_COONH_4_ solutions, respectively, and values were detected by inductively coupled plasma emission spectrum (ICP-OES, iCAP 6000, USA). The ratio of carbon to nitrogen was calculated based on the total carbon and nitrogen content which were determined using an Elementar (Vario Micro, Elememtar Analysensysteme GmbH). The nitrate nitrogen and ammonia nitrogen in soils were extracted by potassium chloride solution and determined by spectrophotometer (UV-1800 Spectrophotometer, Shimadzu Scientific Instruments Inc.). The relevant data of the above discussed tests is listed in Table S1.

Soil microbial DNA was extracted by PowerSoil^®^ DNA Isolation Kit (MoBio Laboratories, USA) according to the instructions. A Nanodrop spectrophotometer (ND-1000, NanoDrop Technologies, USA) was deployed to analyze the quality and concentration of extracted DNA. The fungal ITS1 gene was amplified by primer pair ITS1F (5′-CTTGGTCATTTAGAGGAAGTAA-3′) and ITS2 (5′-GCTGCGTTCTTCATCGATGC-3′)^54^. PCR amplifications were firstly conducted in a total volume of 25 μl using KOD FX Neo (TOYOBO, Japan) according to the manufacturer’s instructions. Briefly, the PCR reactions were amplified by thermocycling (15 cycles at 95 °C for 1 min, 50 °C for 1 min and 72 °C for 1 min) after initialization at 95 °C for 5 min, followed by 7 min final elongation at 72 °C. The PCR products from the first step PCR were purified through VAHTSTM DNA Clean Beads (Vazyme, China). A second round PCR was then performed in a 40 μl solution containing 20 μl 2× Phusion HF MM, 8 μl ddH_2_O, 10 μM of each primer and 10 μl PCR products from the first step. Thermal cycling conditions were as follows: an initial denaturation at 98 °C for 30 s, followed by 10 cycles at 98 °C for 10 s, 65 °C for 30 s min and 72 °C for 30 s, with a final extension at 72 °C for 5 min. Finally, all PCR products were quantified by Quant-iT™ dsDNA HS Reagent (Thermo Scientific, USA) and pooled together. The PCR products were mixed with the same volume of 2× loading buffer, and electrophoresis was performed on a 1.8% agarose gel for detection. Samples with a bright main strip of approximately 450 bp were chosen and mixed in equidensity ratios. Then, a mixture of PCR products was purified using a GeneJET Gel Extraction Kit (Thermo Scientific, USA). Sequencing libraries were validated using an Agilent 2100 Bioanalyzer (Agilent Technologies, United States), and quantified using a Qubit Fluorometer (Qubit 2.0, Thermo Scientific, USA). Finally, paired-end sequencing was conducted using an Illumina HiSeq 2500 platform (Illumina Inc., USA) at Biomarker Bioinformatics Technology Co., Ltd. (Beijing, China). The generated sequences were submitted in the Sequence Read Archive (SRA) database of NCBI under accession number PRJNA781129.

FLASH V1.2.11 software was used to merge the paired-end reads with default settings^55^. The raw sequences data was quality filtered using the Trimmomatic V0.33^56^, and the parameter setting was as follows: Window size as 50 bp, and the reads will be cut from the start of the window once average Q-score within the window is lower than 20. Then chimera sequences were detected using the UCHIME V8.1 with default settings^57^. The resultant sequences that shared at least 97% similarity were clustered into OTUs by using the USEARCH V10.0^58^, and conservative threshold for OTU filtration is 0.005%^59^. Taxonomic classifications were conducted using RDP classifier V2.2 with a confidence threshold of 80%^60^ using the UNITE V7.2 database^61^. The α-diversity of soil fungal community, include Chao 1, Shannon, and Faith’s PD indexes, was calculated by QIIME2^62^. Across these indexes, OTUs and Chao 1 counts for species richness, while Shannon and Faith’s PD reflects species diversity. The RDA was performed using vegan package in R^63^. Pearson’s correlation analysis was conducted according to the abundance and variation of each species in each sample, and data with correlation greater than 0.2 and *p* value less than 0.05 were screened to build correlation network by Python V3.7.3. The heatmap was created using connections between the relative abundance of fungal species and tobacco leaf quality. The analyses were constructed using the top 60 most abundant species of soil fungal community and displayed at the significant of *p* < 0.05 level. Pearson correlations used to assess the associations between the chemical components and environmental factors were performed using SPSS 20.0. The figures of THS and heatmap of tobacco quality and environmental factors were produced using Origin Pro 2017.

### Statement

The collection of the cultivated tobacco samples was done by getting the permission from the local suppliers. The authors confirm that all methods were performed in accordance with the relevant guidelines and regulations.

## Supplementary Information

Supplementary Information.

## Abbreviations

RSN: Ratio of reducing sugar to nicotine
RNN: Ratio of nitrogen to nicotine
RKCl: Ratio of potassium to chloride
THS: Total harmony scores
OM: Organic matter
AvK: Available potassium
AvP: Available phosphorous
RDA: Redundancy analysis
OTUs: Operational taxonomic units
