# Supplementary material for: Soil fungal communities affect the chemical quality of flue-cured tobacco leaves in Bijie, Southwest China
Source: Sci Rep. 2022 Feb 18;12:2815. doi: 10.1038/s41598-022-06593-x (PMC8857190; doi:10.1038/s41598-022-06593-x)
Supplement: Supplementary file 1 — Supplementary Information. [file 41598_2022_6593_MOESM1_ESM.docx]

**Supplement data**

**Table.S1** Information of sampling sites and properties of soil samples. “AvP” and “AvK” means the available phosphorus and available potassium of soils, “C/N ratio” represent the ratio of total carbon content to total nitrogen content.

| Sample ID | Longitude | Latitude | Soil type | Elevation  m a.s.l. | C/N ratio | pH | AvP  mg/kg | AvK  mg/kg | Organic matter  g/kg | Sand  % | Silt  % | Clay % | Ammonia  mg/kg | Nitrate  mg/kg |  |
| --- | --- | --- | --- | --- | --- | --- | --- | --- | --- | --- | --- | --- | --- | --- | --- |
|  |  |  |  |  |  |  |  |  |  |  |  |  |  |  |  |
| S1 | 27.02N | 105.92E | yellow soil | 1,304 | 9.37 | 7.11±0.09 | 88.2±5.36 | 487±1.48 | 48.0±4.02 | 6.71 | 57.1 | 42.9 | 5.21±0.95 | 45.3±0.74 |  |
| S2 | 27.06N | 105.83E | yellow soil | 1,395 | 8.74 | 5.78±0.04 | 93.2±6.88 | 492±2.12 | 46.3±6.18 | 14.3 | 69.4 | 30.6 | 41.5±3.17 | 46.6±0.44 |  |
| S3 | 27.30N | 106.20E | yellow soil | 1,129 | 10.6 | 5.72±0.06 | 45.1±4.21 | 178±11.6 | 40.2±0.60 | 4.56 | 79.6 | 20.4 | 11.1±1.29 | 45.4±2.09 |  |
| S4 | 26.43N | 105.62E | yellow soil | 1,452 | 11.2 | 6.14±0.10 | 20.8±2.23 | 312±26. 5 | 56.0±6.24 | 12.7 | 68.5 | 31.5 | 10.4±1.86 | 22.1±4.21 |  |
| S5 | 26.68N | 106.05E | yellow soil | 1,295 | 9.30 | 7.43±0.28 | 60.4±5.24 | 319±15.6 | 62.3±5.63 | 15.8 | 65.2 | 34.8 | 5.22±1.04 | 46.0±1.29 |  |
| S6 | 26.72N | 106.11E | yellow soil | 1,331 | 2.27 | 5.48±0.08 | 109±0.52 | 301±17.0 | 68.9±2.89 | 10.4 | 79.3 | 20.7 | 6.60±1.48 | 47.1±0.60 |  |
| S7 | 27.36N | 106.46E | yellow soil | 942 | 9.54 | 5.82±0.19 | 24.9±3.38 | 190±17.1 | 42.4±1.28 | 15.9 | 57.3 | 42.8 | 9.88±0.88 | 37.5±3.18 |  |
| S8 | 27.57N | 106.06E | yellow soil | 1,351 | 8.40 | 4.35±0.16 | 62.7±5.76 | 267±15.1 | 22.4±3.24 | 8.25 | 74.9 | 25.1 | 37.4±1.37 | 43.8±1.83 |  |
| S9 | 26.89N | 105.07E | yellow brown soil | 1,735 | 15.2 | 7.89±0.04 | 13.6±1.93 | 162±8.46 | 44.1±4.44 | 24.6 | 63.0 | 37.0 | 3.20±0.65 | 21.0±2.24 |  |
| S10 | 26.91N | 105.37E | yellow soil | 1,558 | 18.5 | 4.55±0.10 | 28.1±2.33 | 180±13.2 | 36.3±1.09 | 8.09 | 51.2 | 48.8 | 4.76±0.33 | 37.3±3.30 |  |
| S11 | 27.33N | 104.96E | yellow soil | 1,679 | 9.01 | 5.41±0.54 | 29.7±3.32 | 184±15.3 | 51.2±3.91 | 17.7 | 68.4 | 31.6 | 24.1±2.31 | 41.0±2.88 |  |
| S12 | 27.38N | 105.31E | yellow soil | 1,552 | 9.24 | 4.88±0.50 | 63.0±5.79 | 385±29.1 | 55.5±3.13 | 26.7 | 78.3 | 21.7 | 21.6±2.27 | 45.3±0.45 |  |
| S13 | 27.64N | 105.33E | yellow soil | 1,627 | 13. 6 | 7.75±0.10 | 70.1±5.33 | 150±5.89 | 50.8±3.01 | 4.98 | 55.4 | 39.6 | 7.51±0.85 | 42.6±0.70 |  |
| S14 | 27.35N | 106.05E | yellow soil | 1,361 | 7.84 | 5.33±0.11 | 43.5±4.37 | 290±11.1 | 48.1±2.46 | 11.7 | 78.2 | 21.8 | 49.4±0.67 | 47.9±1.18 |  |
| S15 | 27.21N | 104.19E | yellow brown soil | 1,999 | 12.8 | 5.00±0.09 | 53.5±4.07 | 81.0±4.17 | 80.3±4.99 | 22.5 | 79.3 | 20.7 | 5.11±1.52 | 30.0±3.09 |  |
| S16 | 27.19N | 104.00E | yellow brown soil | 1,894 | 9.79 | 6.05±0.31 | 22.1±1.66 | 449±44.7 | 52.8±5.71 | 10.0 | 52.4 | 47.6 | 22.4±2.84 | 42.1±2.15 |  |
| S17 | 27.22N | 103.83E | yellow soil | 1,579 | 6.73 | 4.61±0.09 | 21.8±2.25 | 239±15.7 | 23.4±3.82 | 4.37 | 82.5 | 17.5 | 78.5±7.36 | 43.6±4.04 |  |
| S18 | 27.04N | 103.88E | yellow brown soil | 2,104 | 9.92 | 5.22±0.03 | 19.8±3.6 | 86.4±7.99 | 17.7±3.09 | 32.3 | 89.1 | 10.9 | 3.35±0.55 | 40.5±4.70 |  |
| S19 | 26.91N | 103.96E | yellow brown soil | 2,099 | 10.8 | 5.80±0.01 | 10.3±1.56 | 203±12.5 | 98.1±11.5 | 3.74 | 53.4 | 42. 9 | 17.5±1.72 | 34.4±3.36 |  |
| S20 | 26.85N | 104.19E | yellow brown soil | 2,152 | 9.15 | 5.35±0.27 | 16.3±1.21 | 130±3.39 | 44.5±2.95 | 7.36 | 55.3 | 44.8 | 18.0±1.99 | 25.2±0.67 |  |

**Table S2** The harmony scores of the eight selected indexes. “RNN” represent the ratio of total nitrogen to nicotine. “RSN” was the ratio of reducing sugar to nicotine. “RKCl” means the ratio of potassium to chloride.

| ID | Nicotine | Total nitrogen | Reducing sugar | Potassium | Starch | RNN | RSN | RKCl |
| --- | --- | --- | --- | --- | --- | --- | --- | --- |
| S1 | 94.0±10.4 | 64.3±7.67 | 56.3±12.8 | 82.4±0.22 | 87.8±3.4 | 58.2±7.32 | 97.2±2.52 | 100±0 |
| S2 | 83.0±4.6 | 55.5±3.29 | 41.0±10.75 | 84.4±0.18 | 75.2±3.4 | 89.3±2.34 | 48.8±13.9 | 100±0 |
| S3 | 99.1±1.51 | 47.3±3.67 | 24.2±12.3 | 78.7±0.45 | 48.8±3.53 | 55.9±8.9 | 72.5±4 | 100±0 |
| S4 | 84.5±5.65 | 59.2±7.07 | 71.9±14.5 | 81.2±0.26 | 41.6±3.67 | 90.3±6.17 | 68.2±10.7 | 100±0 |
| S5 | 45.0±26.7 | 92.9±6.17 | 95.9±5.00 | 89.2±0.33 | 98.0±1.76 | 61.1±12.1 | 87.7±7.09 | 92.5±1.07 |
| S6 | 18.7±16.4 | 97.4±4.43 | 85.6±5.94 | 88.6±0.25 | 100±0 | 55.3±1.12 | 88.1±4.34 | 94.6±3.49 |
| S7 | 0±0 | 100±0 | 95.0±3.09 | 78.3±0.73 | 97.8±1.80 | 50.0±1.71 | 65.3±8.2 | 100±0 |
| S8 | 39.8±3.34 | 50.6±5.06 | 39.7±12.9 | 92.4±0.19 | 66.2±3.24 | 78.7±13.6 | 0±0 | 100±0 |
| S9 | 66.6±8.92 | 59.6±10.43 | 49.2±14.0 | 73.3±0.61 | 32.0±3.77 | 94.2±6.49 | 26.6±16.1 | 100±0 |
| S10 | 75.0±13 | 100±0 | 99.1±1.59 | 84.8±0.17 | 87.6±3.17 | 79.7±8.35 | 89.6±5.09 | 100±0 |
| S11 | 100±0 | 62.7±9.08 | 62.8±9.02 | 73.3±0.42 | 48.4±3.68 | 68.19±8.7 | 93.7±3.24 | 100±0 |
| S12 | 33.0±13.6 | 98.2±3.16 | 87.6±3.72 | 93.6±0.3 | 85.4±3.77 | 61.5±5.33 | 90.0±3.76 | 100±0 |
| S13 | 100±0 | 58.6±5.57 | 92.0±5.06 | 82.6±0.27 | 17.4±3.63 | 59.0±7.3 | 100±0 | 100±0 |
| S14 | 24.0±17.5 | 93.0±6.11 | 77.4±13.8 | 100±0 | 99.1±1.61 | 76.8±13.95 | 91.8±1.94 | 100±0 |
| S15 | 100±0 | 100±0 | 42.7±6.7 | 79.33±0.45 | 94.6±1.82 | 90.2±5.62 | 92.0±1.78 | 19.12±0.29 |
| S16 | 53.0±23.7 | 80±10.7 | 22.3±11.7 | 83.6±0.2 | 62.6±3.7 | 56.7±9.72 | 98.4±2.42 | 95.2±0.41 |
| S17 | 5.50±5.17 | 100±0 | 94. 9±5 | 63.5±0.26 | 100±0 | 61.0±2.28 | 78.9±5.22 | 92.1±1.82 |
| S18 | 95.3±8.13 | 97.2±4.79 | 100±0 | 70.7±0.1 | 100±0 | 81.99±2.83 | 90.3±4.90 | 92.7±1.98 |
| S19 | 97.0±2.75 | 68.7±10.4 | 60.1±12.3 | 64.5±0.49 | 92.2±1.67 | 86.59±3.99 | 81.5±3.07 | 34.3±0.74 |
| S20 | 79.5±4.37 | 52.9±3.55 | 60.1±14.6 | 70.7±0.4 | 60.0±3.44 | 87.9±8.10 | 53.4±10.7 | 61.4±1.32 |

**Table S3** The standard for grading chemical composition compatibility of flue-cured tobacco *(27, 28)*. Between the change range of each chemical component content and the corresponding score range is a linear relationship.

| Indexes | Scores | | | | | |
| --- | --- | --- | --- | --- | --- | --- |
|  | 100.00 | 100.00 – 90.00 | 90.00 – 80.00 | 80.00 – 70.00 | 70.00 – 60.00 | < 60.00 |
| Reducing sugar (%) | 18.00 – 22.00 | 18.00 – 16.00  22.00 – 24.00 | 16.00 – 14.00  24.00 – 26.00 | 14.00 – 13.00  26.00 – 27.00 | 13.00 – 12.00  27.00 – 28.00 | < 12.00  > 28.00 |
| Nicotine (%) | 2.20 – 2.80 | 2.20 – 2.00  2.80 –2.90 | 2.00 – 1.80  2.90 – 3.00 | 1.80 – 1.70  3.00 – 3.10 | 1.70 – 1.60  3.10 – 3.20 | < 1.60  > 3.20 |
| Total nitrogen (%) | 2.00 – 2.50 | 2.00 – 1.90  2.50 – 2.60 | 1.90 – 1.80  2.60 – 2.70 | 1.80 – 1.70  2.70 – 2.80 | 1.70 – 1.60  2.80 – 2.90 | < 1.60  > 2.90 |
| Potassium (%) | ≥ 2.50 | 2.50 – 2.00 | 2.00 – 1.50 | 1.50 – 1.20 | 1.20 – 1.00 | < 1.00 |
| Starch (%) | < 3.50 | 3.50 – 4.50 | 4.50 – 5.00 | 5.00 – 5.50 | 5.50 – 6.00 | ≥ 6.00 |
| Total nitrogen to nicotine ratio | 0.95 – 1.05 | 0.95 – 0.80  1.05 – 1.20 | 0.80 – 0.70  1.20 – 1.30 | 0.70 – 0.65  1.30 – 1.35 | 0.65 – 0.60  1.35 – 1.40 | < 0.60  > 1.40 |
| Reducing sugar to nicotine ratio | 8.50 – 9.50 | 8.50 – 7.00  9.50 – 12.00 | 7.00 – 6.00  12.00 – 13.00 | 6.00 – 5.50  13.00 – 14.00 | 5.50 – 5.00  14.00 –15.00 | < 5.00  > 15.00 |
| Potassium to chloride ratio | ≥ 8.00 | 8.00 – 6.00 | 6.00 – 5.00 | 5.00 – 4.50 | 4.50 – 4.00 | < 4.00 |

**References:**

27. X. Wang, et al., Study on the primary chemical components, sensory quality of flue-cured tobacco and their correlativity in Bijie. Journal of Henan Agricultural Sciences 41, 58-61,64 (2012). (in Chinese)

28. S. Zhang, et al., Analysis of variation characteristics and coordination of conventional chemical components of flue-cured tobacco in Youyang County，Chongqing City. Acta Agriculturae Jiangxi 32, 75-86 (2020). (in Chinese)


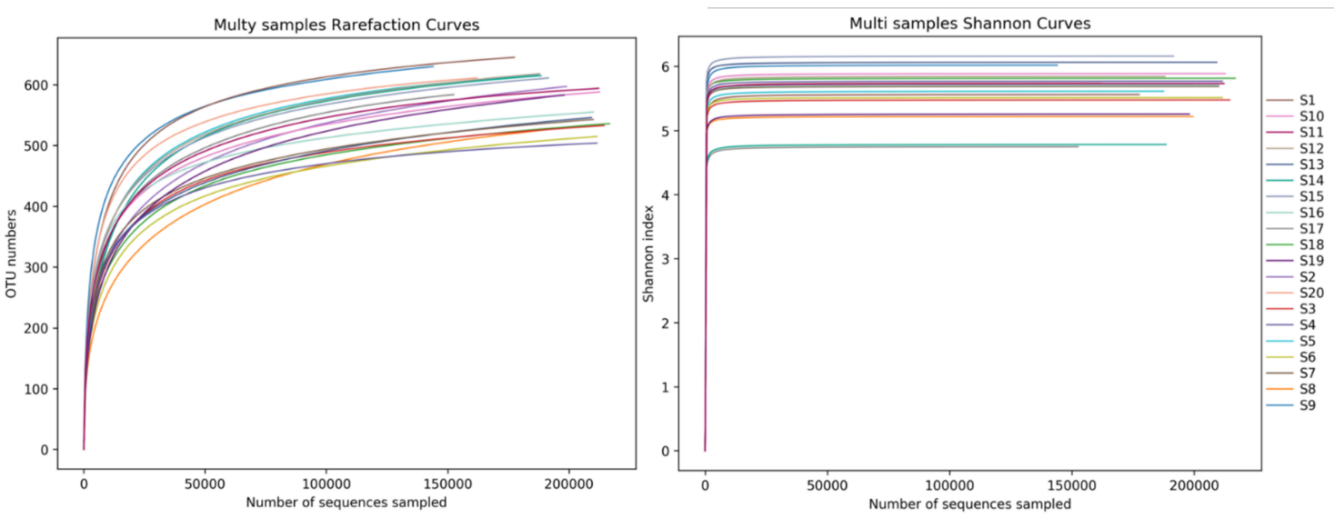


(A)

(B)

**Fig. S1** Rarefaction curves (a) and Shannon curves (b) of the samples.


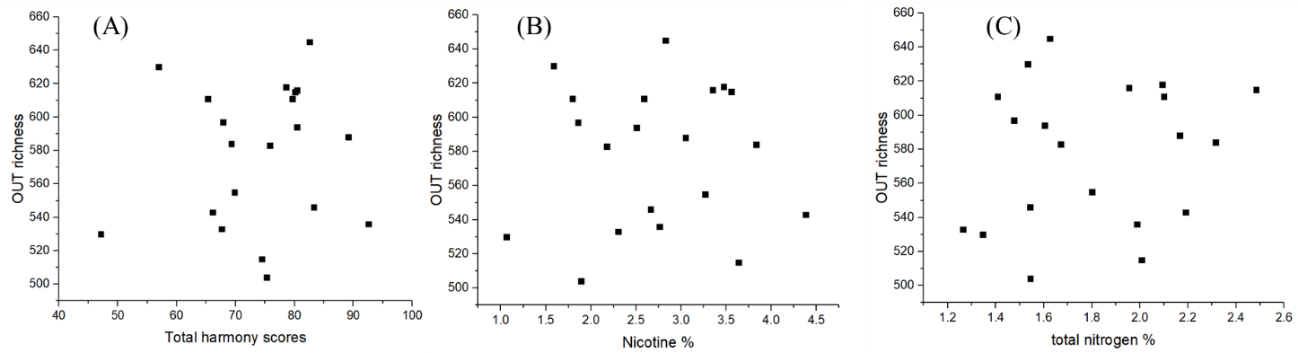


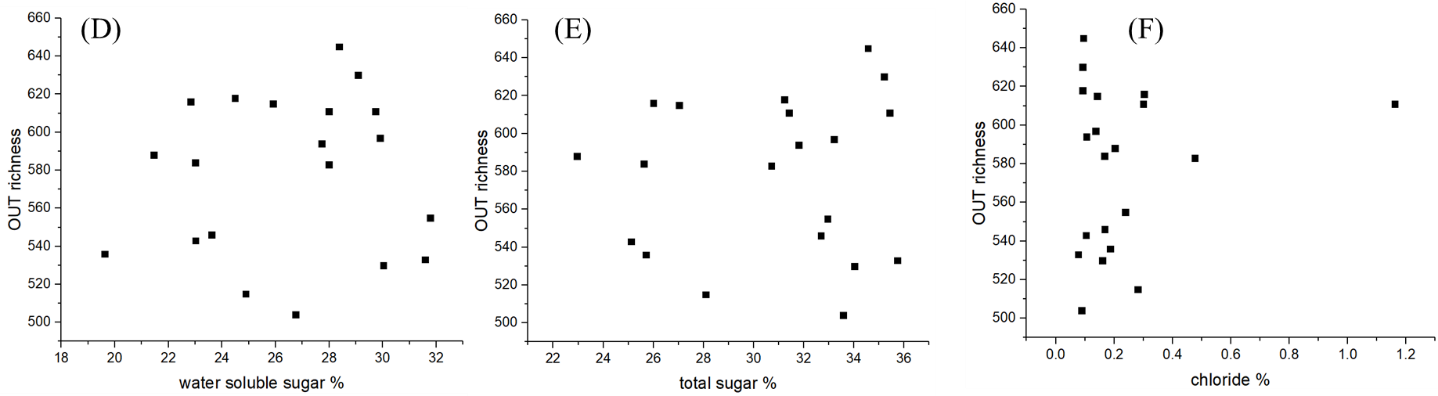


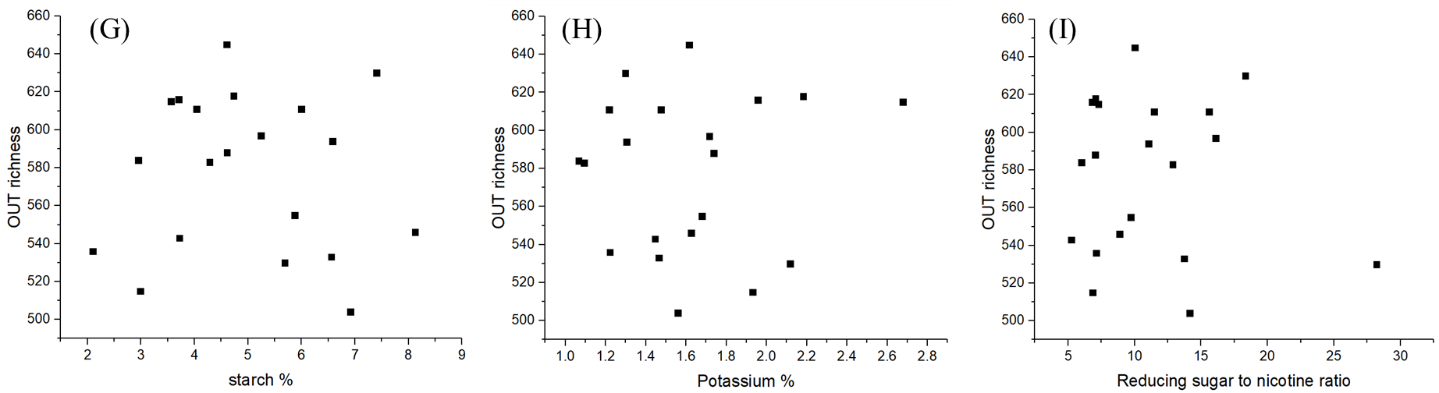


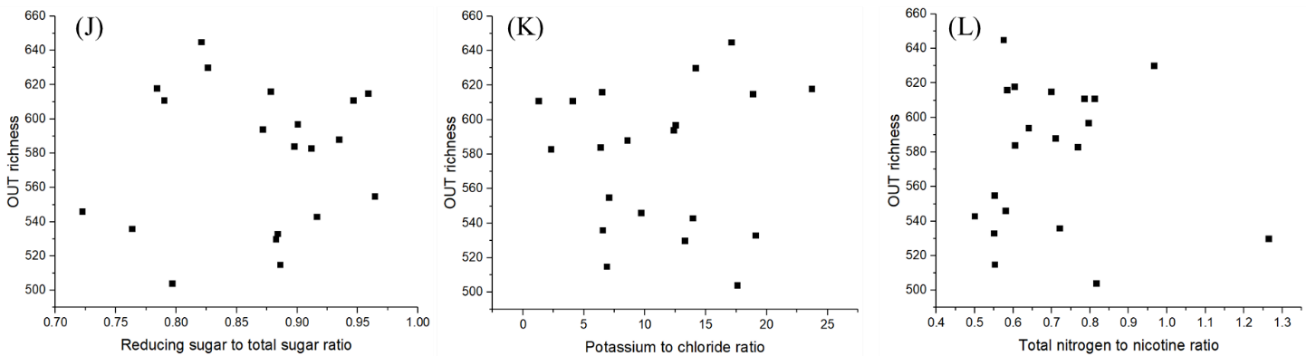


**Fig. S2** Scatter diagram of total harmony scores (a), chemical components (nicotine (b), total nitrogen (c), reducing sugar (d), total sugar (e), chloride (f), starch (g), and potassium (h)), ratio of chemical contents (reducing sugar to nicotine ratio (i), reducing sugar to total sugar ratio (j), potassium to chloride ratio (k), and total nitrogen to nicotine ratio (l)) with OTUs richness of soil fungi.
